# Supplementary figures and images for: Toxoplasma gondii virulence factor ROP1 reduces parasite susceptibility to murine and human innate immune restriction
Source: PLoS Pathog. 2022 Dec 7;18(12):e1011021. doi: 10.1371/journal.ppat.1011021 (PMC9762571; doi:10.1371/journal.ppat.1011021)

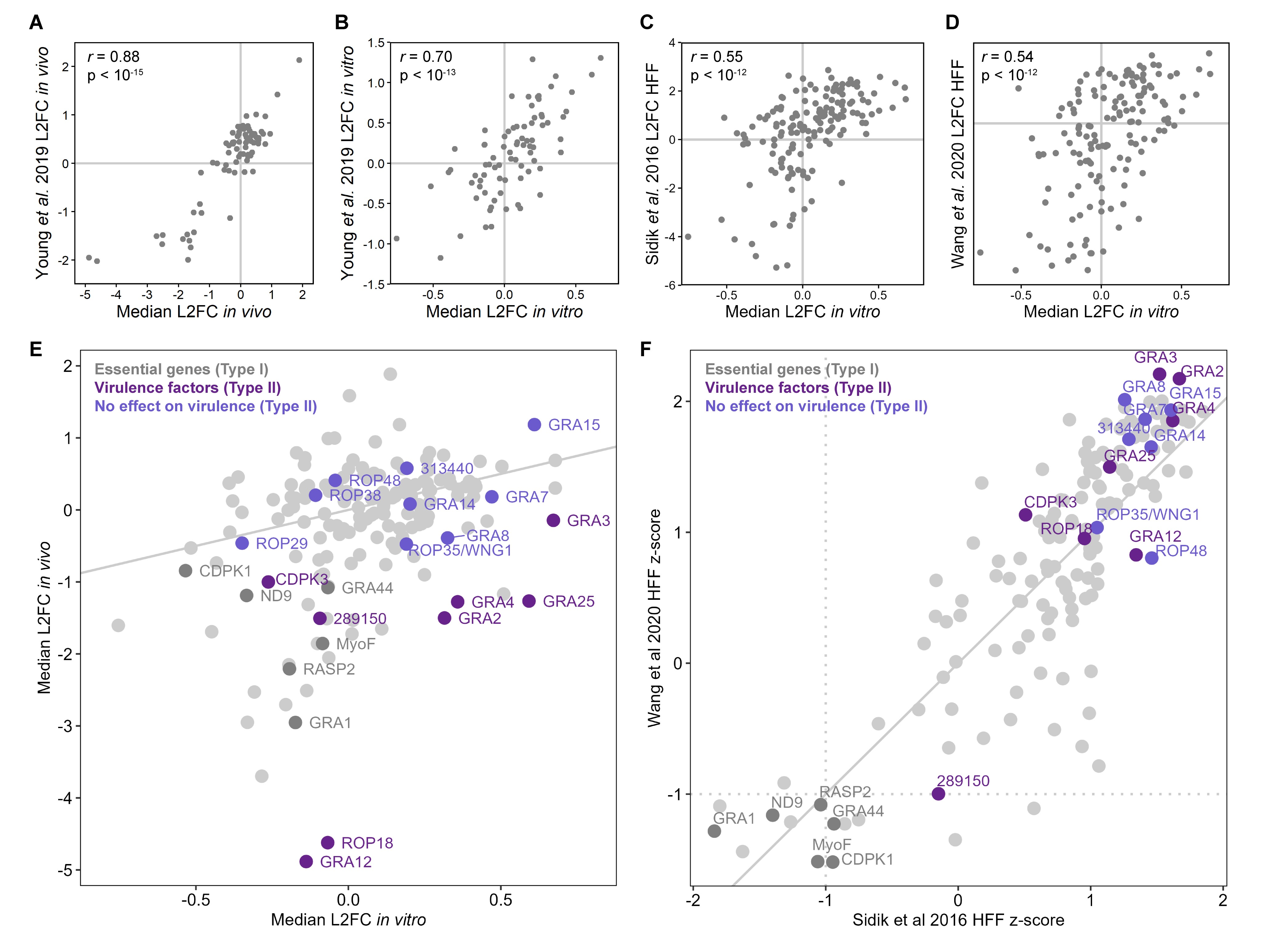

Supplement: S1 Fig — A. Correlation between in vivo L2FCs from this study and in vivo L2FCs from [15]. r = Pearson’s product-moment correlation coefficient. B, C, D. Correlation between in vitro L2FCs from this study and B in vitro L2FCs from [15], C HFF passage 3 L2FCs from [18], and D HFF passage 8 L2FCs from [19]. r = Pearson’s product-moment correlation coefficient. E. Scatter plot of median L2FCs for each gene in vitro and in vivo. Control genes for which knockouts have previously been tested for an effect on virulence in Type II strains of T. gondii are labelled. Genes which have been found to be essential in Type I strains of T. gondii are also labelled. The grey line indicates equal in vitro and in vivo L2FCs. F. Z-score-transformed HFF L2FCs from [18] and [19] for genes screened in this study. The grey line indicates equal z-scores in both studies and dotted lines indicate z-scores of -1 in each study. (TIF) [file ppat.1011021.s001.tif]

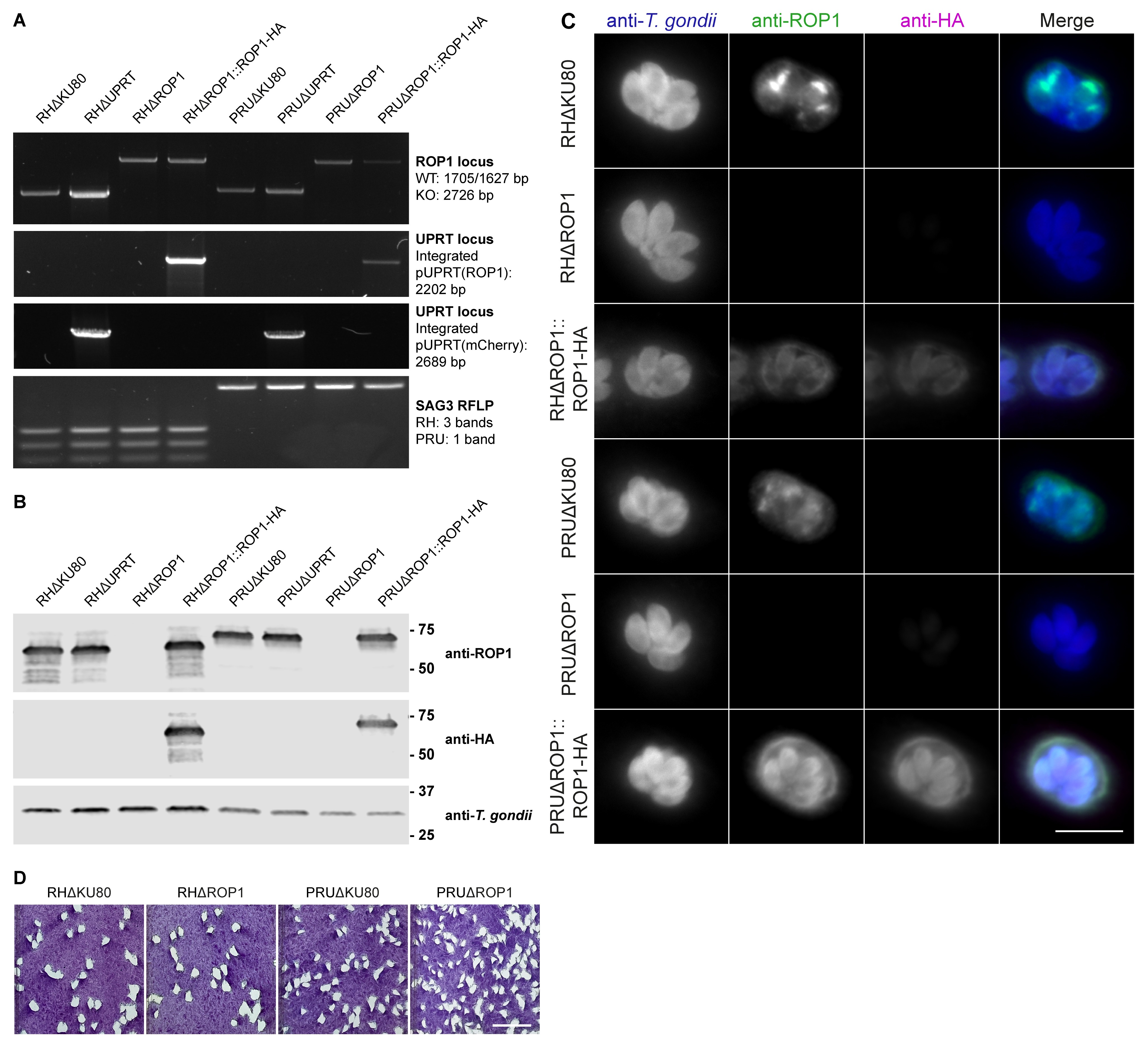

Supplement: S2 Fig — A. Verification of correct integration of knockout and complementation constructs by diagnostic PCR and verification of strain genotype by restriction fragment length polymorphism (RFLP) of the SAG3 gene [61]. Knockouts were obtained by integration of an mCherry-T2A-HXGPRT linear PCR cassette facilitated by co-transfection with a Cas9-sgRNA plasmid targeting the gene of interest. For ROP1 complementation, the ROP1 coding sequence and native promoter were cloned from RHΔKU80 or PRUΔKU80 genomic DNA into the pUPRT vector, adding a single C-terminal HA tag, linearised and integrated by double homologous recombination following co-transfection with a Cas9-sgRNA plasmid targeting the UPRT locus. B. Verification of ROP1 and ROP1-HA expression by Western blot. C. Immunofluorescence verification of ROP1 knockout and complemented T. gondii cell lines using 1 minute permeabilisation. Scale bar = 10 μm. D. Plaques formed by RHΔKU80, RHΔROP1, PRUΔKU80 and PRUΔROP1 parasites after seven days’ growth in a monolayer of HFFs. Scale bar = 1 cm. (TIF) [file ppat.1011021.s002.tif]

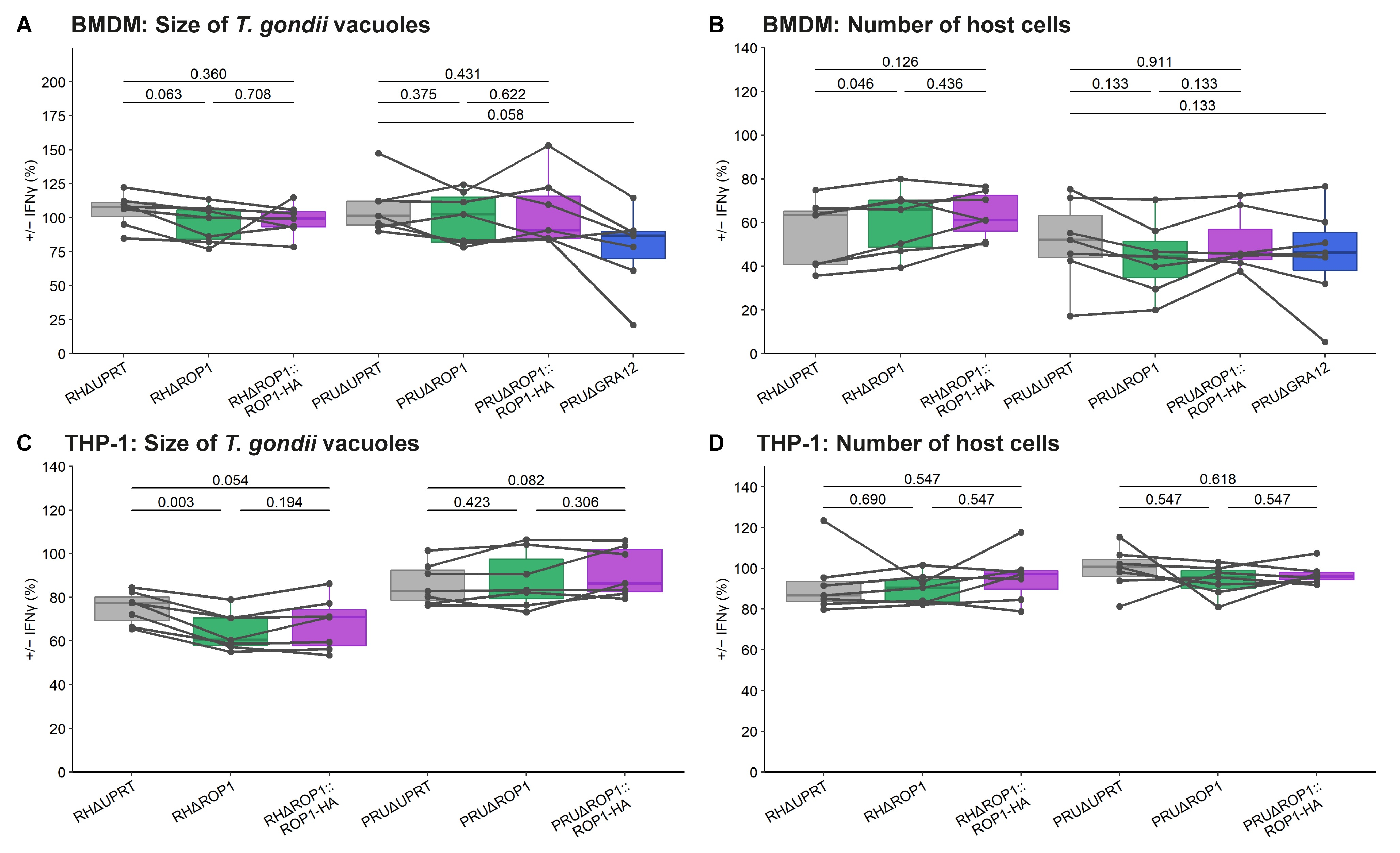

Supplement: S3 Fig — A, B. IFNγ-dependent growth restriction of T. gondii in BMDMs. BMDMs were stimulated with IFNγ for 24 h, infected with T. gondii cell lines for a further 24 h and parasite growth quantified by automated fluorescence imaging and analysis. A T. gondii vacuole size (mean parasites per vacuole) in IFNγ-stimulated BMDMs is shown as a percentage of the size in unstimulated BMDMs. B The number of IFNγ-stimulated host BMDM nuclei is shown as a percentage of the number of unstimulated BMDM nuclei. p-values were calculated by paired two-sided t-test with Benjamini-Hochberg adjustment. C, D. IFNγ-dependent growth restriction of T. gondii in THP-1-derived macrophages. Differentiated THP-1 macrophages were stimulated with IFNγ, infected, and parasite growth quantified as above. C T. gondii vacuole size (mean parasites per vacuole) in IFNγ-stimulated THP-1 macrophages is shown as a percentage of the size in unstimulated macrophages. D The number of IFNγ-stimulated host THP-1 macrophage nuclei is shown as a percentage of the number of unstimulated macrophage nuclei. p-values were calculated by paired two-sided t-test with Benjamini-Hochberg adjustment. (TIF) [file ppat.1011021.s003.tif]

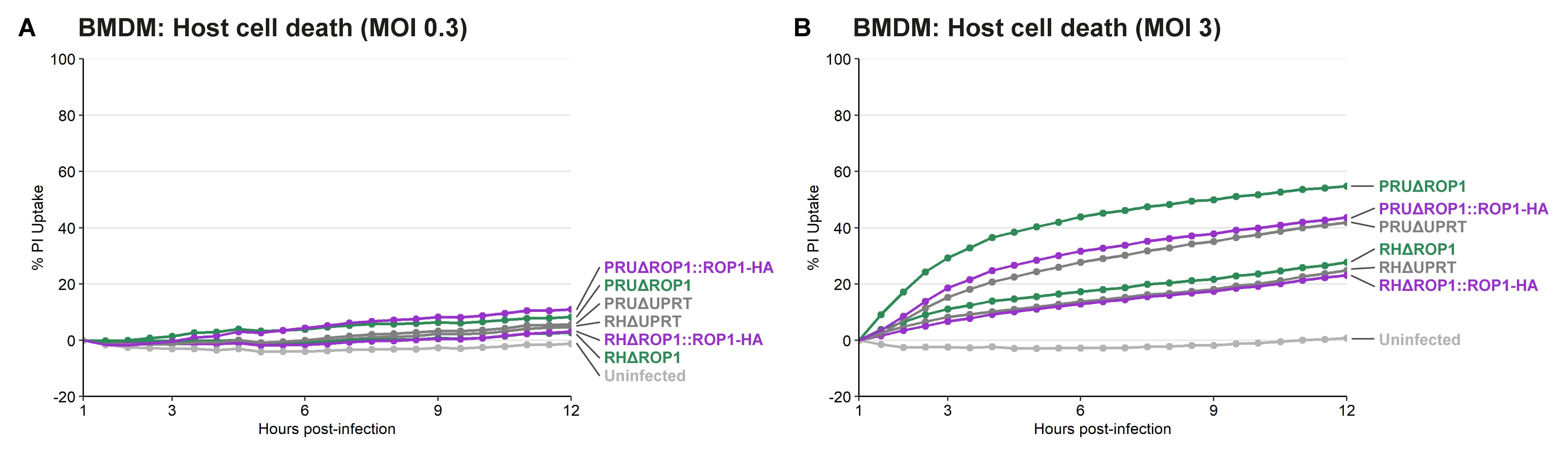

Supplement: S4 Fig — A, B. Propidium iodide uptake of IFNγ-stimulated BMDMs infected at an MOI of A 0.3 or B 3. Propidium iodide fluorescence was measured every 30 minutes from 1–12 hours post-infection. Curves represent the mean of five replicates. Uptake at 12 hours post-infection is shown in Fig 3. (TIF) [file ppat.1011021.s004.tif]

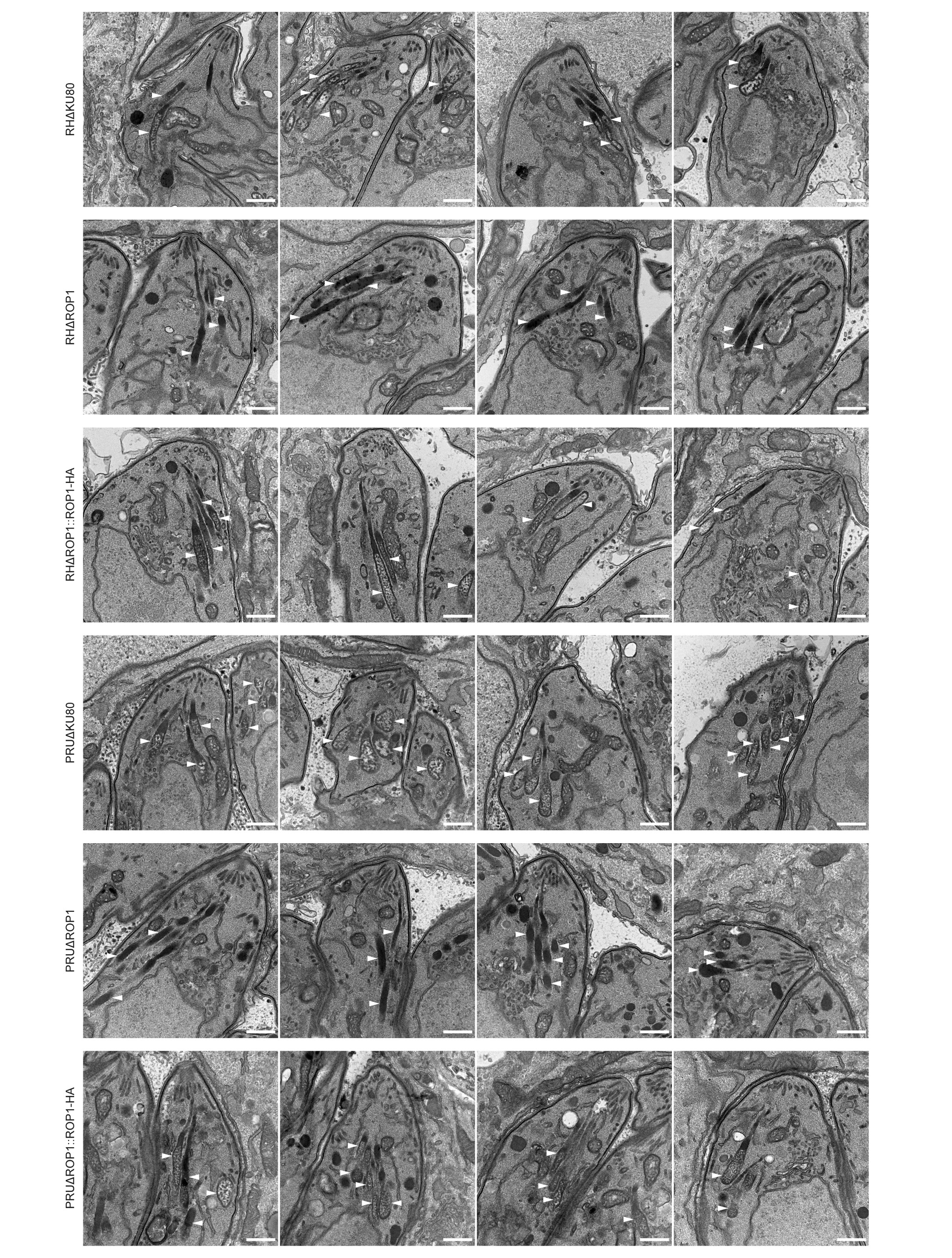

Supplement: S5 Fig — Scale bar = 500 μm. (TIF) [file ppat.1011021.s005.tif]

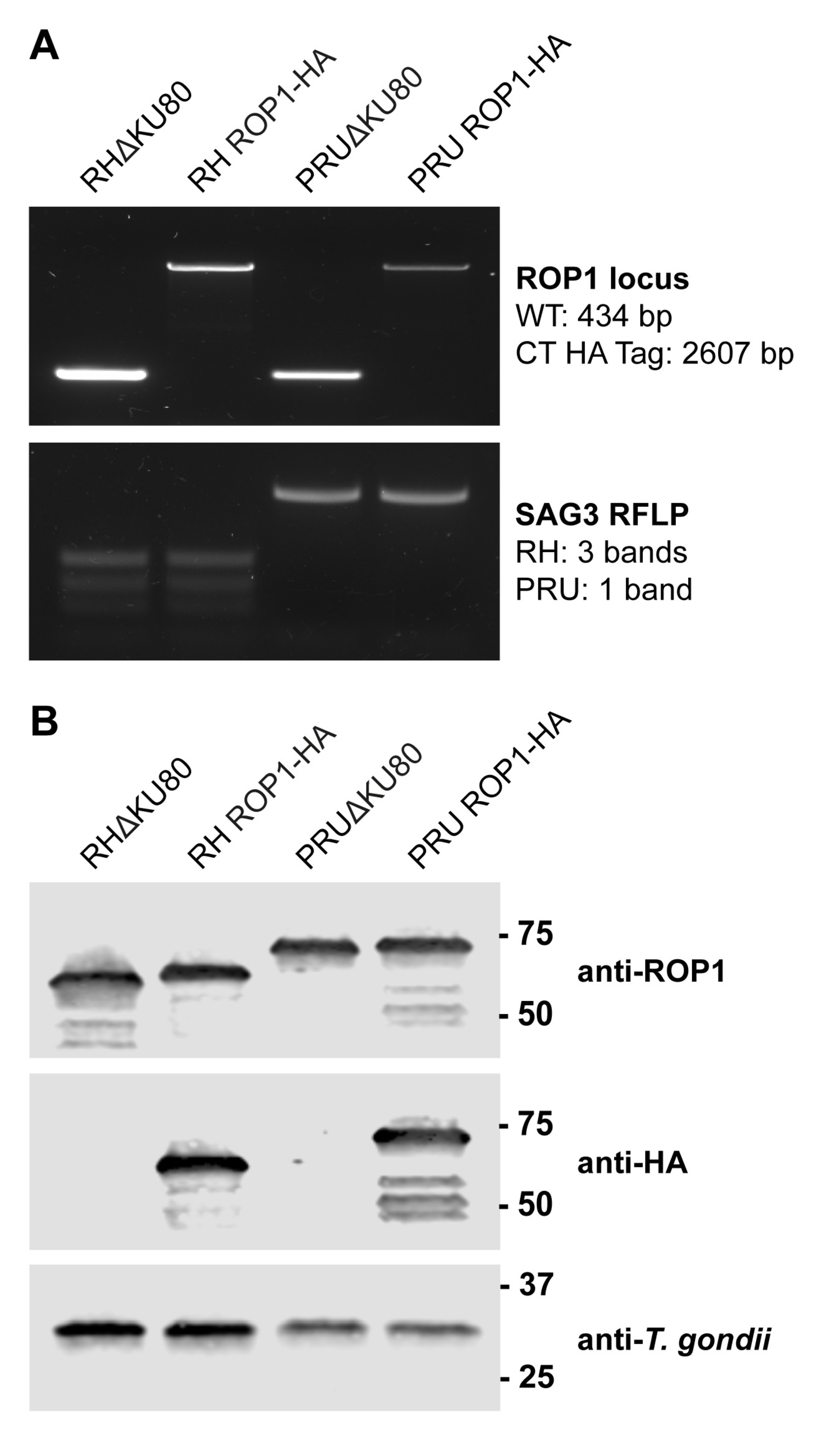

Supplement: S6 Fig — A. Verification of correct integration of C-terminal HA-tagging construct by diagnostic PCR and verification of strain genotype by restriction fragment length polymorphism (RFLP) of the SAG3 gene [61]. HA-tagged cell lines were obtained by double homologous recombination with an HA-HXGPRT linear PCR cassette facilitated by co-transfection with a Cas9-sgRNA plasmid targeting the 3’ UTR of ROP1. Clonal T. gondii cell lines were obtained by limiting dilution. B. Verification of ROP1 and ROP1-HA expression by Western blot. (TIF) [file ppat.1011021.s006.tif]

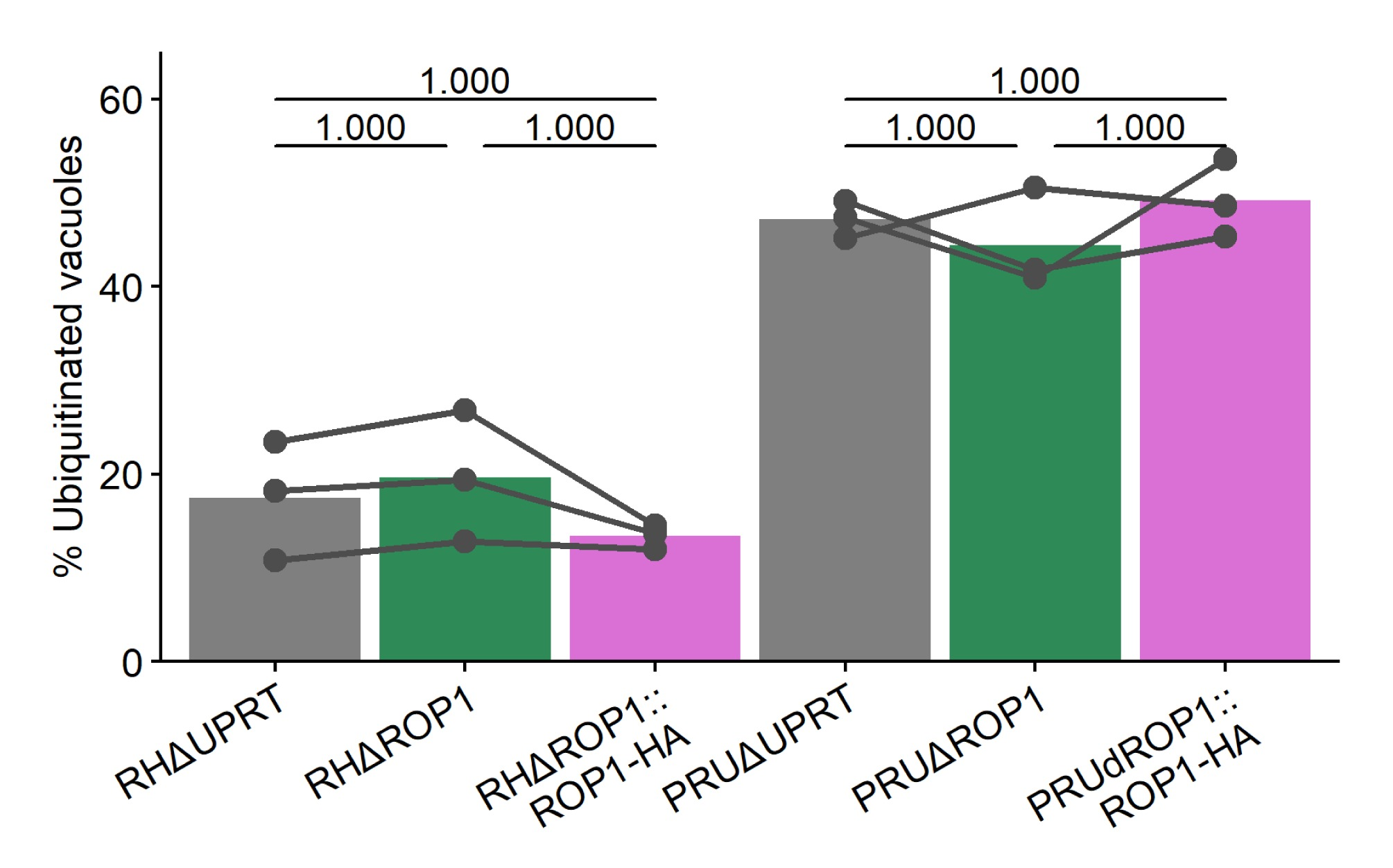

Supplement: S7 Fig — BMDMs were stimulated with 100 U/mL IFNγ for 24 h, infected for 3 h, fixed, and stained with an anti-ubiquitinylated proteins antibody. The percentage of ubiquitinated vacuoles was quantified manually from blinded immunofluorescence microscopy images. p-values were calculated by paired two-sided t-test with Benjamini-Hochberg adjustment. (TIF) [file ppat.1011021.s007.tif]

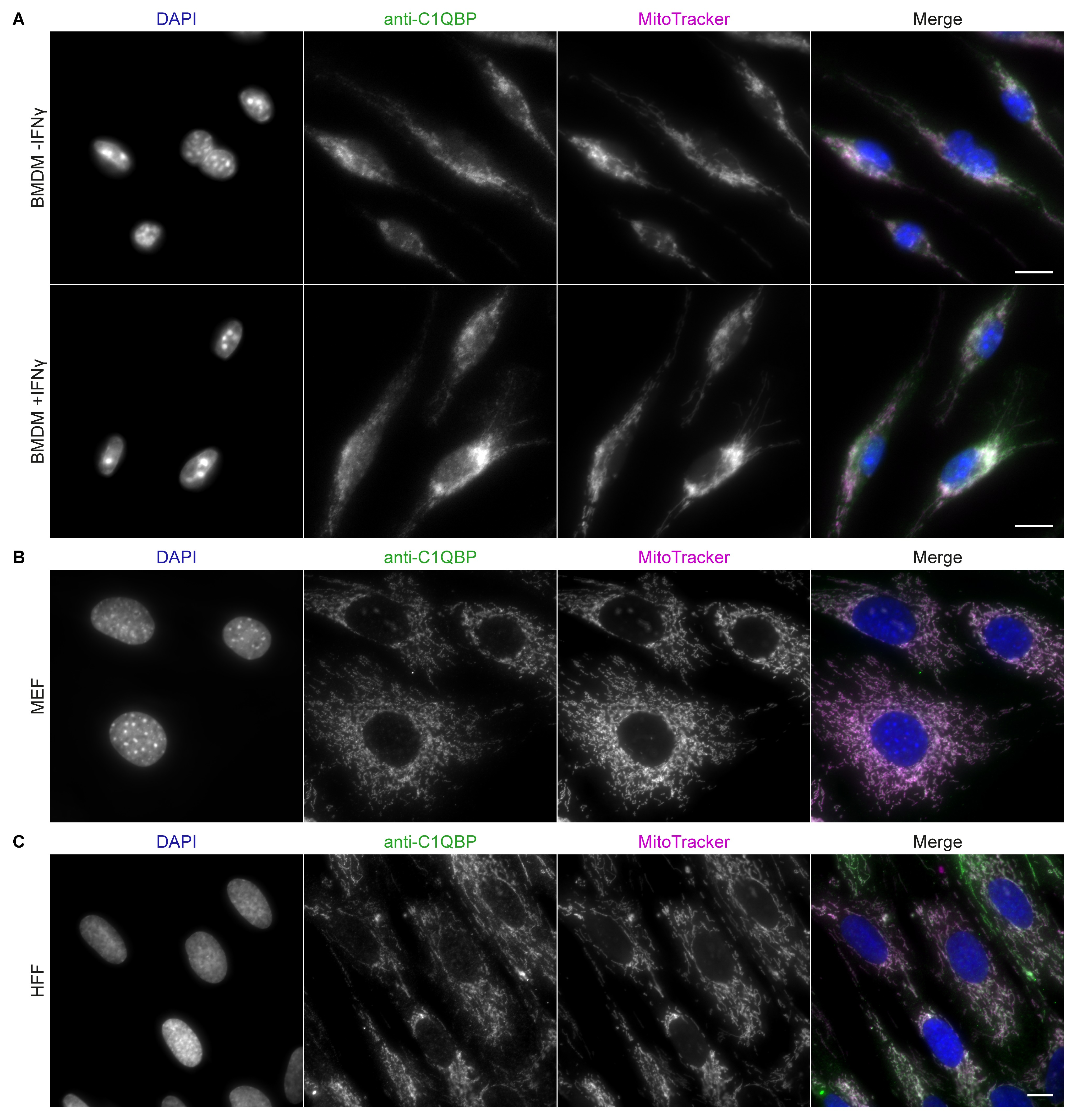

Supplement: S8 Fig — A, B, C. Immunofluorescence localisation of C1QBP in A C57BL/6J BMDMs stimulated +/- 100 U/mL IFNγ for 24 h, B primary C57BL/6J MEFs, and C HFFs. Scale bars = 10 μm. (TIF) [file ppat.1011021.s008.tif]

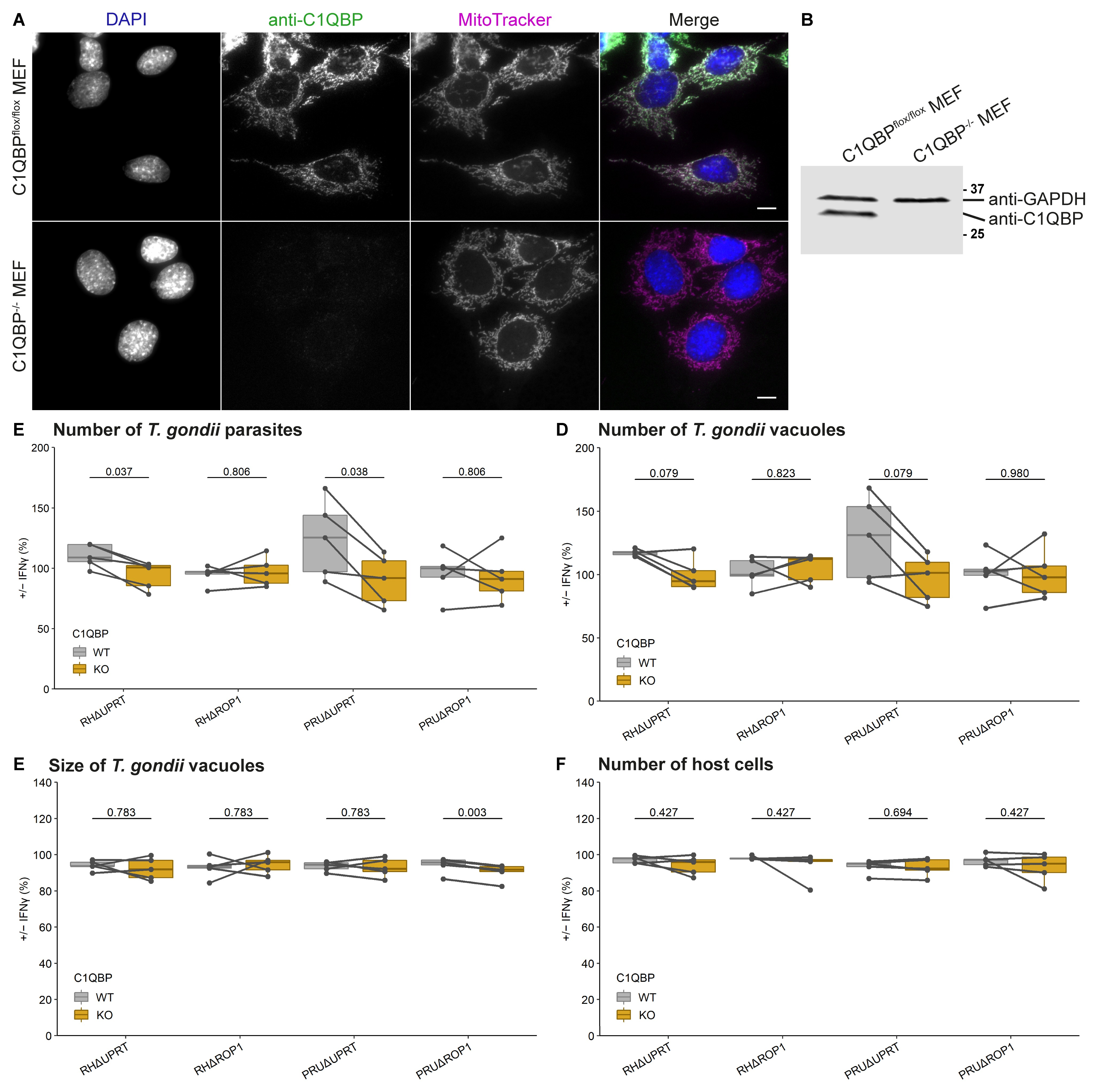

Supplement: S9 Fig — A. Validation of C1QBPflox/flox and C1QBP-/- immortalised MEFs by immunofluorescence assay. Scale bar = 10 μm. B. Validation of C1QBPflox/flox and C1QBP-/- immortalised MEFs by Western blot. C, D, E, F. IFNγ-dependent growth restriction of T. gondii in C1QBPflox/flox (WT) and C1QBP-/- (KO) immortalised MEFs. MEFs were stimulated with IFNγ for 24 h, infected with T. gondii cell lines for a further 24 h, and parasite growth quantified by automated fluorescence imaging and analysis. Parasite growth in IFNγ-stimulated BMDMs is shown as a percentage of that in unstimulated BMDMs in terms of C total number of T. gondii parasites, D number of T. gondii vacuoles, E vacuole size, and E number of host cells. p-values were calculated by paired two-sided t-test with Benjamini-Hochberg adjustment. (TIF) [file ppat.1011021.s009.tif]
