# Supplementary material for: Toxoplasma gondii virulence factor ROP1 reduces parasite susceptibility to murine and human innate immune restriction
Source: PLoS Pathog. 2022 Dec 7;18(12):e1011021. doi: 10.1371/journal.ppat.1011021 (PMC9762571; doi:10.1371/journal.ppat.1011021)
Supplement: S7 Data — (PDF) [file ppat.1011021.s016.pdf]

Table. Analysis sequence of Harmony v5.0 to determine Toxoplasma within THP-1

Step	Building block	Input / Method / Output
1	Input Image	Channel group:1, Sequences: All, Flatfield Correction: Basic, Brightfield Correction, Stack Processing: Maximum Projection
2	Find Image Region	Channel: Alexa 647, ROI: None / Method: Common Threshold, Threshold: 0.1, Split into Objects, Area: > 100px^2 / Output Population: Image Region, Output Region: Image Region
3	Filter Image	Channel: DAPI / Method: Smoothing, Filter: Median, Scale: 4 px / Output Image: Median Smoothed
4	Find Nuclei	Channel: Median Smoothed, ROI: None / Method: C, Common Threshold: 0.1, Area: > 20 µm^2, Splitting Coefficient: 10.5, Individual Threshold: 0.5, Contrast: > 0.1 / Output Population: Nuclei
5	Calculate Intensity Properties	Channel: DAPI, Population: Nuclei, Region: Nucleus / Method: Standard Mean / Property Prefix: Intensity Nucleus DAPI
6	Calculate Intensity Properties (2)	Channel: mCherry, Population: Nuclei, Region: Nucleus / Method: Standard Mean / Property Prefix: Intensity Nucleus mCherry
7	Select Population	Population: Nuclei / Method: Filter by Property, Intensity Nucleus mCherry mean: <= 800, Intensity Nucleus DAPI Mean: > 300, Boolean Operations: F1 and F2 / Output Population: Nuclei Selected
8	Find Cytoplasm	Channel: Alexa 647, Nuclei: Nuclei Selected, Method: A, Individual Threshold: 0.1
9	Filter Image (2)	Channel: mCherry / Method: Smoothing, Filter: Gaussian, Scale: 7 px / Output Image: Gaussian Smoothed toxo
10	Find Spots	Channel: Gaussian Smoothed toxo, ROI: Nuclei Selected, ROI Region: Cell / Method: A, Relative Spot Intensity: > 0.03, Splitting Sensitivity: 1, Calculate Spot Properties / Output Population: Vacuole candidate
11	Calculate Intensity Properties (3)	Channel: mCherry, Population: Vacuole candidate, Region: Spot / Method: Standard, Mean / Property Prefix: Intensity Spot mCherry
12	Select Population (2)	Population: Vacuole candidate / Method: Filter by Property, Intensity Spot mCherry Mean: > 1200 / Output Population: Vacuole
13	Find Spots (2)	Channel: DAPI, ROI: Vacuole, ROI Region: Spot / Method: C, Radius: <= 5 px, Contrast: > 0.1, Uncorrected Spot to Region Intensity: > 1, Distance: >= 3px, spot Peak Radius: 0 px, Calculate Spot Properties / Output Population: Toxoplasma
14	Calculate Properties	Population: Vacuole, Method: By Related Population, Related Population: Toxoplasma, Number of Toxoplasma / Property Suffix: per vacuole
15	Calculate Properties (2)	Population: Nuclei Selected / Method: By Related Population, Related Population: Vacuole, Number of Vacuole, Spot Area [px^2]: Mean, Number of Toxoplasma- per vacuole: Mean / Property Suffix: per Cell
16	Select Population (3)	Population: Nuclei Selected / Method: Filter by Property, Number of Vacuole- per cell: >= 1 / Output Population: Infected cell
17	Define Results	Method: List of Outputs<br>Population: Toxoplasma (Number of Objects)<br>Population: Nuclei Selected (Number of Objects, Infected cell: Mean)<br>Population: Vacuole (Number of Objects, Number of Toxoplasma- per vacuole: Mean)<br>Population: Infected cell (Number of Objects, Number of Vacuole- per Cell: Mean, Spot Area [px^2]- Mean per Cell: Mean, Number of Toxoplasma- per vacuole - Mean per Cell: Mean)

		Method: Formula Output<br>Formula: a/b<br>Population Type: Objects<br>Variable a: Toxoplasma - Number of Objects<br>Variable b: Infected cell - Number of Objects<br>Output Name: mean toxo number per infected cell

Table. Analysis sequence of Harmony v5.0 to determine Toxoplasma within MEF

Step	Building block	Input / Method / Output
1	Input Image	Channel group:1, Sequences: All, Flatfield Correction: Basic, Brightfield Correction, Stack Processing: Maximum Projection
2	Find Image Region	Channel: Alexa 647, ROI: None / Method: Common Threshold, Threshold: 0.1, Split into Objects, Area: > 100px^2 / Output Population: Image Region, Output Region: Image Region
3	Filter Image	Channel: DAPI / Method: Smoothing, Filter: Median, Scale: 6 px / Output Image: Median Smoothed
4	Find Nuclei	Channel: Median Smoothed, ROI: None / Method: C, Common Threshold: 0.4, Area: > 40 µm^2, Splitting Coefficient: 15.1, Individual Threshold: 0.5, Contrast: > 0.1 / Output Population: Nuclei
5	Calculate Intensity Properties	Channel: DAPI, Population: Nuclei, Region: Nucleus / Method: Standard Mean / Property Prefix: Intensity Nucleus DAPI
6	Calculate Intensity Properties (2)	Channel: mCherry, Population: Nuclei, Region: Nucleus / Method: Standard Mean / Property Prefix: Intensity Nucleus mCherry
7	Select Population	Population: Nuclei / Method: Filter by Property, Intensity Nucleus mCherry mean: <= 700, Intensity Nucleus DAPI Mean: > 500, Intensity Nucleus DAPI Mean: <= 4500, Boolean Operations: F1 and F2 and F3 / Output Population: Nuclei Selected
8	Find Cytoplasm	Channel: Alexa 647, Nuclei: Nuclei Selected, Method: A, Individual Threshold: 0
9	Filter Image (2)	Channel: mCherry / Method: Smoothing, Filter: Gaussian, Scale: 6 px / Output Image: Gaussian Smoothed toxo
10	Find Spots	Channel: Gaussian Smoothed toxo, ROI: Nuclei Selected, ROI Region: Cell / Method: B, Relative Spot Intensity: > 0.8, Splitting Sensitivity: 1, Calculate Spot Properties / Output Population: Vacuole candidate
11	Calculate Intensity Properties (3)	Channel: mCherry, Population: Vacuole candidate, Region: Spot / Method: Standard, Mean / Property Prefix: Intensity Spot mCherry
12	Select Population (2)	Population: Vacuole candidate / Method: Filter by Property, Intensity Spot mCherry Mean: > 320 / Output Population: Vacuole
13	Find Spots (2)	Channel: DAPI, ROI: Vacuole, ROI Region: Spot / Method: C, Radius: <= 5 px, Contrast: > 0.1, Uncorrected Spot to Region Intensity: > 1, Distance: >= 3px, spot Peak Radius: 0 px, Calculate Spot Properties / Output Population: Toxoplasma
14	Calculate Properties	Population: Vacuole, Method: By Related Population, Related Population: Toxoplasma, Number of Toxoplasma / Property Suffix: per vacuole
15	Calculate Properties (2)	Population: Nuclei Selected / Method: By Related Population, Related Population: Vacuole, Number of Vacuole, Spot Area [px^2]: Mean, Number of Toxoplasma- per vacuole: Mean / Property Suffix: per Cell
16	Select Population (3)	Population: Nuclei Selected / Method: Filter by Property, Number of Vacuole- per cell: >= 1 / Output Population: Infected cell
17	Define Results	Method: List of Outputs<br>Population: Toxoplasma (Number of Objects)<br>Population: Nuclei Selected (Number of Objects, Infected cell: Mean)<br>Population: Vacuole (Number of Objects, Number of Toxoplasma- per vacuole: Mean)<br>Population: Infected cell (Number of Objects, Number of Vacuole- per Cell: Mean, Spot Area [px^2]- Mean per Cell: Mean, Number of Toxoplasma- per vacuole - Mean per Cell: Mean)

		Method: Formula Output<br>Formula: a/b<br>Population Type: Objects<br>Variable a: Toxoplasma - Number of Objects<br>Variable b: Infected cell - Number of Objects<br>Output Name: mean toxo number per infected cell

Table. Analysis sequence of Harmony v5.0 to determine Toxoplasma within BMDM

Step	Building block	Input / Method / Output
1	Input Image	Channel group:1, Sequences: All, Flatfield Correction: Basic, Brightfield Correction, Stack Processing: Maximum Projection
2	Find Image Region	Channel: Alexa 647, ROI: None / Method: Common Threshold, Threshold: 0.05, Split into Objects, Area: > 100px^2 / Output Population: Image Region, Output Region: Image Region
3	Filter Image	Channel: DAPI / Method: Smoothing, Filter: Median, Scale: 3 px / Output Image: Median Smoothed
4	Find Nuclei	Channel: Median Smoothed, ROI: None / Method: C, Common Threshold: 0.4, Area: > 20 µm^2, Splitting Coefficient: 7.0, Individual Threshold: 0.4, Contrast: > 0.1 / Output Population: Nuclei
5	Calculate Intensity Properties	Channel: DAPI, Population: Nuclei, Region: Nucleus / Method: Standard Mean / Property Prefix: Intensity Nucleus DAPI
6	Calculate Intensity Properties (2)	Channel: mCherry, Population: Nuclei, Region: Nucleus / Method: Standard Mean / Property Prefix: Intensity Nucleus mCherry
7	Select Population	Population: Nuclei / Method: Filter by Property, Intensity Nucleus mCherry mean: <= 1000, Intensity Nucleus DAPI Mean: > 800, Boolean Operations: F1 and F2 / Output Population: Nuclei Selected
8	Find Cytoplasm	Channel: Alexa 647, Nuclei: Nuclei Selected, Method: D, Individual Threshold: 0.05
9	Filter Image (2)	Channel: mCherry / Method: Smoothing, Filter: Gaussian, Scale: 5 px / Output Image: Gaussian Smoothed toxo
10	Find Spots	Channel: Gaussian Smoothed toxo, ROI: Nuclei Selected, ROI Region: Cell / Method: B, Relative Spot Intensity: > 0.7, Splitting Sensitivity: 1, Calculate Spot Properties / Output Population: Vacuole candidate
11	Calculate Intensity Properties (3)	Channel: mCherry, Population: Vacuole candidate, Region: Spot / Method: Standard, Mean / Property Prefix: Intensity Spot mCherry
12	Calculate Intensity Properties (4)	Channel: DAPI, Population: Vacuole candidate, Region: Spot / Method: Standard, Median / Property Prefix: Intensity Spot DAPI
13	Select Population (2)	Population: Vacuole candidate / Method: Filter by Property, Intensity Spot mCherry Mean: > 700, Intensity Spot DAPI Median: >= 200 / Output Population: Vacuole
14	Find Spots (2)	Channel: mCherry, ROI: Vacuole, ROI Region: Spot / Method: C, Radius: <= 4.5 px, Contrast: > 0.1, Uncorrected Spot to Region Intensity: > 1, Distance: >= 3px, spot Peak Radius: 0 px, Calculate Spot Properties / Output Population: Toxoplasma
15	Calculate Properties	Population: Vacuole, Method: By Related Population, Related Population: Toxoplasma, Number of Toxoplasma / Property Suffix: per vacuole
16	Calculate Properties (2)	Population: Nuclei Selected / Method: By Related Population, Related Population: Vacuole, Number of Vacuole, Spot Area [px^2]: Mean, Number of Toxoplasma- per vacuole: Mean / Property Suffix: per Cell
17	Select Population (3)	Population: Nuclei Selected / Method: Filter by Property, Number of Vacuole- per cell: >= 1 / Output Population: Infected cell
18	Define Results	Method: List of Outputs<br>Population: Toxoplasma (Number of Objects)<br>Population: Nuclei Selected (Number of Objects, Infected cell: Mean)<br>Population: Vacuole (Number of Objects, Number of Toxoplasma- per vacuole: Mean)

		Population: Infected cell (Number of Objects, Number of Vacuole- per Cell: Mean, Spot Area [px²]- Mean per Cell: Mean, Number of Toxoplasma- per vacuole - Mean per Cell: Mean)<br><br>Method: Formula Output<br>Formula: a/b<br>Population Type: Objects<br>Variable a: Toxoplasma - Number of Objects<br>Variable b: Infected cell - Number of Objects<br>Output Name: mean toxo number per infected cell

Table. Analysis sequence of Harmony v5.0 to determine IRGB6 recruited Toxoplasma within BMDM

Step	Building block	Input / Method / Output
1	Input Image	Channel group:1, Sequences: All, Flatfield Correction: Basic, Brightfield Correction, Stack Processing: Maximum Projection
2	Find Image Region	Channel: Alexa 647, ROI: None / Method: Common Threshold, Threshold: 0.05, Split into Objects, Area: > 100px^2 / Output Population: Image Region, Output Region: Image Region
3	Filter Image	Channel: DAPI / Method: Smoothing, Filter: Median, Scale: 3 px / Output Image: Median Smoothed
4	Find Nuclei	Channel: Median Smoothed, ROI: None / Method: C, Common Threshold: 0.05, Area: > 30 μm^2, Splitting Coefficient: 7.0, Individual Threshold: 0.4, Contrast: > 0.1 / Output Population: Nuclei
5	Calculate Intensity Properties	Channel: DAPI, Population: Nuclei, Region: Nucleus / Method: Standard Mean / Property Prefix: Intensity Nucleus DAPI
6	Calculate Intensity Properties (2)	Channel: mCherry, Population: Nuclei, Region: Nucleus / Method: Standard Mean / Property Prefix: Intensity Nucleus mCherry
7	Select Population	Population: Nuclei / Method: Filter by Property, Intensity Nucleus mCherry mean: <= 750, Intensity Nucleus DAPI Mean: > 1000, Boolean Operations: F1 and F2 / Output Population: Nuclei Selected
8	Find Cytoplasm	Channel: Alexa 647, Nuclei: Nuclei Selected, Method: D, Individual Threshold: 0.35
9	Find Spots	Channel: Gaussian Smoothed toxo, ROI: Nuclei Selected, ROI Region: Cell / Method: B, Relative Spot Intensity: > 0.3, Splitting Sensitivity: 1, Calculate Spot Properties / Output Population: Vacuole candidate
10	Calculate Intensity Properties (3)	Channel: mCherry, Population: Vacuole candidate, Region: Spot / Method: Standard, Mean / Property Prefix: Intensity Spot mCherry
11	Calculate Intensity Properties (4)	Channel: DAPI, Population: Vacuole candidate, Region: Spot / Method: Standard, Median / Property Prefix: Intensity Spot DAPI
12	Select Population (2)	Population: Vacuole candidate / Method: Filter by Property, Intensity Spot mCherry Mean: > 1200, Intensity Spot DAPI Median: >= 250, Spot Area [px^2]: > 20 / Output Population: Vacuole
13	Select Region	Population: Vacuole, Region: Spot / Method: Resize Region [µm/px], Outer Border: -4 px, Restrictive Population: None, Restrictive Region: empty, Inner Border: 2 px / Output Region: Vacuole Resized
14	Calculate Intensity Properties (5)	Channel: Alexa 488, Population: Vacuole, Region: Vacuole Resized / Method: Standard, Median / Property Prefix: Intensity Vacuole Resized Alexa 488
15	Calculate Intensity Properties (6)	Channel: Alexa 488, Population: Nuclei Selected, Region: Cytoplasm / Method: Standard, Median / Property Prefix: Intensity Cytoplasm Alexa 488
16	Calculate Properties	Population: Vacuole, Method: By Related Population, Related Population: Nuclei Selected, Intensity Cytoplasm Alexa 488 Median: Mean / Property Suffix: per Object
17	Calculate Properties (2)	Population: Vacuole, Method: By Formula, Formula: A/B, Variable A; Intensity Vacuole Resized Alexa 488 Median, Variable B: Intensity Cytoplasm Alexa 488 Median- Mean per Object / Output Property: IRG Ratio Vacuole/Cell
18	Select Population (3)	Population: Vacuole / Method: Filter by Property, IRG Ratio Vacuole/Cell: > 2.3 / Output Population: IRG recruited vacuole
19	Find Spots (2)	Channel: mCherry, ROI: Vacuole, ROI Region: Spot / Method: C, Radius: <= 4.5 px, Contrast: > 0.1, Uncorrected Spot to Region Intensity: > 1, Distance: >= 3px, spot Peak Radius: 0 px, Calculate Spot Properties / Output Population: Toxoplasma

20	Calculate Properties (3)	Population: Vacuole, Method: By Related Population, Related Population: Toxoplasma, Number of Toxoplasma / Property Suffix: per vacuole
21	Calculate Properties (4)	Population: Nuclei Selected / Method: By Related Population, Related Population: Vacuole, Number of Vacuole, Spot Area [px^2]: Mean, Number of Toxoplasma- per vacuole: Mean / Property Suffix: per Cell
22	Select Population (4)	Population: Nuclei Selected / Method: Filter by Property, Number of Vacuole- per cell: >= 1 / Output Population: Infected cell
23	Calculate Properties (5)	Population: Infected cell / Method: By Related Population, Related Population: IRG recruited vacuole, Number of IRG recruited Vacuole, Intensity Cytoplasm Alexa 488 Median / Property Suffix: per Cell
24	Select Population (5)	Population: Infected cell / Method: Filter by Property, Number of IRG recruited vacuole- per cell: >= 1 / Output Population: IRG recruited Infected cell
25	Define Results	Method: List of Outputs<br>Population: Toxoplasma (Number of Objects)<br>Population: Infected cell (Number of Objects, Number of Vacuole- per Cell: Mean, Spot Area [px^2]- Mean per Cell: Mean, Number of Toxoplasma- per vacuole - Mean per Cell: Mean, Number of IRG recruited vacuole- per Cell: Mean)<br>Population: Nuclei Selected (Number of Objects, Intensity Cytoplasm Alexa 488 Median: Mean, Infected cell: Mean)<br>Population: Vacuole (Number of Objects, Intensity Vacuole Resized Alexa 488 Median: Mean, IRG Ratio Vacuole/Cell: Mean, Number of Toxoplasma- per vacuole: Mean)<br>Population: IRG recruited vacuole (Intensity Vacuole Resized Alexa 488 Median: Mean)<br><br>Method: Formula Output<br>Formula: a/b<br>Population Type: Objects<br>Variable a: Toxoplasma - Number of Objects<br>Variable b: Infected cell - Number of Objects<br>Output Name: mean toxo number per infected cell<br><br>Method: Formula Output<br>Formula: a/b*100<br>Population Type: Objects<br>Variable a: IRG recruited vacuole - Number of Objects<br>Variable b: Vacuole - Number of Objects<br>Output Name: % of IRG recruited vacuole in total vacuole<br><br>Method: Formula Output<br>Formula: a/b*100<br>Population Type: Objects<br>Variable a: IRG recruited Infected cell - Number of Objects<br>Variable b: Infected cell - Number of Objects<br>Output Name: % IRG recruited vacuole cells in infected cells
