# Supplementary material for: Toxoplasma gondii virulence factor ROP1 reduces parasite susceptibility to murine and human innate immune restriction
Source: PLoS Pathog. 2022 Dec 7;18(12):e1011021. doi: 10.1371/journal.ppat.1011021 (PMC9762571; doi:10.1371/journal.ppat.1011021)
Supplement: Attachment — Submitted filename: 125493_1_rebuttal_2455716_rhs4nx.pdf [file ppat.1011021.s018.pdf]

Manuscript number: RC-2022-01374R

Corresponding author(s): Treeck, Moritz

[Please use this template only if the submitted manuscript should be considered by the affiliate journal as a full revision in response to the points raised by the reviewers.

If you wish to submit a preliminary revision with a revision plan, please use our "Revision Plan" template. ***It is important to use the appropriate template to clearly inform the editors of your intentions.****]*

1. General Statements [optional]

This section is optional. Insert here any general statements you wish to make about the goal of the study or about the reviews.

In this work, we set out to identify effector proteins of *T. gondii* that contribute to parasite immune evasion through pooled CRISPR knockout screening *in vivo*, taking advantage of our recently developed tools (Young et al 2019) and the recent identification of a large number of previously unstudied secreted proteins (Barylyuk et al 2020). Among other hits, which will be of interest to the field and merit further investigation, we identified ROP1, the first-identified rhoptry protein of *T. gondii* whose function has remained unknown to date. We showed that ROP1 inhibits interferon-gamma-mediated innate immune restriction of both the RH and PRU strains of *T. gondii*, which is unusual for a rhoptry effector protein. Furthermore, we found that ROP1 inhibits restriction in both murine and human macrophages, implicating an as-yet unidentified conserved mechanism of parasite restriction.

In the discussion section, the reviewers agreed on four major points which we have addressed as follows:

- *In vivo* validation of ROP1. We have now carried out mouse infections using the PRUΔKU80, PRUΔROP1, and complemented lines, showing that PRUΔROP1 is completely avirulent. This matches our *in vivo* CRISPR screen and *in vitro* IFNγ restriction assays results, and confirms that ROP1 is an important *T. gondii* virulence factor. We highlight the discrepancy with Soldati et al 1995, and suggest that this important role of ROP1 was previously overlooked in *T. gondii* RH due to the "hypervirulence" of this strain in laboratory mice.
- Clarify discrepancies in Irgb6 recruitment compared to published data: We revisited our image analysis pipeline for this data and corrected an error in the host cell

segmentation step which was causing erroneously high calling of Irgb6 recruitment. The recruitment we now measure is now less variable and is consistent with published data, confirming that ROP1 does not affect Irgb6 recruitment or rhoptry bulb protein secretion.

- Conduct specific assays to measure host cell death or remove claims about this. We have carried out kinetic propidium iodide uptake assays as suggested by Reviewer #3. These have clarified that there is minimal parasite-induced host cell death at low MOI which cannot explain the host cell loss observed in the high-content imaging restriction assays. At an MOI of 0.3, ROP1 has no detectable effect on host cell death, while at a much higher MOI of 3, ROP1 knockout moderately increases cell death of PRU-infected BMDMs. Since cell death in macrophages is reported to result from exposure of parasite-derived PAMPS to host cytosolic sensors, we suggest that this increased host cell death at high MOI is a secondary effect of vacuole disrustion. Alternatively, these findings raise the interesting possibility of a differential phenotype for ROP1 at low versus high parasite burden.

- Queries regarding the restriction assays in C1QBP$^{flox/flox}$/$^{-/-}$ MEFs. We included these data as we found statistically significant differences between the C1QBP$^{flox/flox}$ and C1QBP$^{-/-}$ MEFs that were dependent on the presence of ROP1 in the parasites. However, given the concerns raised by all three reviewers regarding the apparent lack of *T. gondii* restriction in these cells, we have withdrawn all conclusions relating to the putative role of C1QBP in parasite restriction. The data are included now in a supplementary figure only as a reference for other researchers working on the role of C1QBP in innate immunity who may use these previously published cell lines. We have made additional attempts to explore the link between ROP1 and C1QBP that have been unsuccessful, which are now mentioned in the discussion section. Although the co-immunoprecipitation of C1QBP with ROP1 is interesting, given the putative role of C1QBP in regulating autophagy and innate immune responses, further exploration of this potential interaction will require new tools and substantially more work that is beyond the scope of this manuscript.

Further responses to comments raised by individual reviewers and details of further revisions are described below.

2. Point-by-point description of the revisions

This section is mandatory. Please insert a point-by-point reply describing the revisions that were already carried out and included in the transferred manuscript.

We thank all reviewers for their input and suggestions.

Reviewer #1

Major Comments

The authors identified ROP1 as a significant hit from their in vivo screen. However, they have not done any validation experiments using rop1 KO parasites in mice. Previous studies have shown no virulence defect in mice for rop1 KO in the type I background (PMID: 8719248). The result could be different in the type II strain used here, but this needs to be tested and shown.

Soldati et al 1995 (PMID: 8719248) demonstrated that there was no virulence defect associated with ROP1 knockout in the RH strain. However, the RH strain is extremely virulent in most laboratory mice strains, which can mask phenotypes observed in a type II strain. We have now carried out *in vivo* infections of C57BL/6J mice using the type II Prugniaud strain, and have shown a severe virulence defect for PRUΔROP1 parasites which is rescued by complementation. This matches our *in vivo* CRISPR screen and *in vitro* IFNγ restriction assays results, and confirms that ROP1 is a virulence factor *in vivo*.

The data in Fig. 2 and S3 do support that reduced parasitemia was due to decrease in number of vacuoles rather than their size or host cell death. However, it is important to control for invasion and/or egress differences of rop1 KO parasites in IFN-g activated cells.

Soldati et al 1995 (PMID: 8719248) demonstrated that ROP1 has no role in invasion in HFFs, therefore it would be very surprising if ROP1 were to have a specific IFNγ-dependent role in invasion in macrophages. There is no involvement of rhoptry bulb-localised proteins in the predominant model of invasion, only rhoptry neck and microneme proteins.

"Natural" egress after ~48 h would not occur here as the cells are fixed after 24 h. IFNγ-induced "early" egress has been documented in HFFs, A549s, and macrophages *in vivo*, and is apparent through increased host cell death/lysis (Niedelman et al 2013 PMID: 24042117, Rinkenberger et al 2021 PMID: 34871166, Tomita et al 2009 PMID: 19846885). We have now carried out prodium iodide uptake assays to more accurately quantify parasite-induced host cell death, and find no differences

between strains at an MOI of 0.3, the targeted MOI we use in our restriction assays. At a higher MOI of 3, we find a moderate increase in host cell death in PRUΔROP1-infected BMDMs, which we suggest results from increased exposure of parasite-derived PAMPs to cytosolic sensors (Fisch et al 2019 PMID: 31268602, Zhao et al 2009 PMID: 19197351). Alternatively, this increased host cell death could result from increased rates of early egress, or from direct inhibition of programmed cell death pathways.

It is important and informative to depict absolute parasite/size when making multiple comparisons. For example, the data in Fig. 4E shows C1QBP-/- MEFs can clear both RH and PRU better than the WT. However the authors do not comment on what is the meaning of > 100% parasite numbers in IFN-g treated MEFs with respect to untreated in WT. Since the data are normalized, it is difficult to appreciate what the actual differences are. Additionally, C1QBP-/- MEFs show close to equal survival in control and IFN-g treated condition (approximately 100%). Is it correct to infer that C1QBP has no effect on parasite survival? This should be considered in light of the comment below on colocalisation of C1QBP.

It is standard practise to show IFNγ-dependent restriction as a percentage of unstimulated cells as this reflects the hypothesis being tested and the statistical tests carried out, as for example in Wang et al 2020 PMID: 33067458, Matta et al 2019 PMID: 31413201, Gay et al 2016 PMID: 27503074, Bando et al 2018 PMID: 30283439, Fleckenstein et al 2012 PMID: 22802726. Absolute numbers are included in the supplementary data for further reference.

The >100% survival observed in the C1QBP$^{flox/flox}$ and C1QBP$^{-/-}$ MEFs is puzzling. We conclude that these cell lines have largely lost the ability to restrict *T. gondii* parasites as a result of the immortalisation process and/or passage history, and what little restriction we observe is at the limit of detection in our assay. MEFs are otherwise known to restrict both RH and PRU parasites (Niedelman et al 2012 PMID: 22761577). We included these data as we found statistically significant differences between the C1QBP$^{flox/flox}$ and C1QBP$^{-/-}$ MEFs which were dependent on the presence of ROP1 in the parasites. However, after the concerns raised by all three reviewers we agree that it is better to not to draw conclusions from these assays given the lack of parasite restriction. We will include these data only in the supplementary figures as a reference for other researchers working on the role of C1QBP in innate immunity who may use these previously published cell lines.

The authors observed good restriction of both RH and PRU in IFN-g activated THP1s without cell death (Fig S1D). It is important to incorporate this information into the main result and discuss their implications in contrast to a previous report from 2019 (Fisch et al, PMID: 31268602).

As mentioned above, we have carried out propidium iodide uptake assays to address questions regarding host cell death more precisely, which are now included as a main figure in the revised manuscript. While Fisch et al measured host cell death during infection of stimulated THP-1s at an MOI of 3, our restriction assays were carried out at a targeted MOI of 0.3. The Howard lab has shown that host cell death is directly proportional to MOI in BMDMs (Zhao et al 2009 PMID: 19197351), and we also find that at an MOI of 0.3 there is little detectable host cell death in BMDMs. While we are not aware of a similar study in THP-1s, it is likely also the case that at low MOIs there is little detectable cell death.

The authors should consider conducting C1QBP functional assays to explore potential roles in parasite survival/growth within host cells. For example, it would be informative to measure the extent of autophagy or transcriptomic profile of the KO to deduce or suggest possible mechanisms of restriction.

All reviewers have raised concerns regarding the restriction assays in the $C1QBP^{flox/flox}$/$C1QBP^{-/-}$ MEFs, particularly that they do not appear to restrict parasite growth as would be expected. As a result, we have decided to withdraw conclusions from these assays regarding the role of C1QBP. For the same reason, we feel that further functional assays using these cells would be of limited value. As an alternative, we attempted siRNA-mediated knockdown of C1QBP in primary BMDMs using a pool of three commercial siRNAs, but were able to achieve only <10% knockdown. Generating our own immortalised BMDM or THP-1 knockout cell lines would require a substantial amount of time and work. We will therefore limit our conclusions regarding C1QBP and state only that it is a potential interaction partner of ROP1.

Minor Comments

The authors state in their text that "C1QBP localised primarily to the mitochondria (Figure S7A) and therefore did not see any co-localisation with ROP1". The authors should discuss in more detail as this finding seems to contradict the interaction studies. Is there any independent evidence to corroborate the interaction studies and show they are not simply an in vitro artifact?

We have now added immunofluorescence images of C1QBP and ROP1 in infected MEFs and HFFs to the main figure and discuss this in further detail. We find that

C1QBP primary localises to the mitochondria, therefore there is some overlap with ROP1 signal at the PVM in RH as type I strains recruit mitochondria to the vacuole via MAF1B (Pernas et al 2014 PMID: 24781109, Adomako-Ankomah et al 2016 PMID: 26920761). However, type II strains do not recruit host mitochondria therefore we see little overlap with ROP1 in PRU. Furthermore, the precise localisation of C1QBP is a matter of some debate, such that it is unclear whether ROP1 and C1QBP are topologically able to interact. One study reported that C1QBP is exclusively localised to the mitochondrial matrix and therefore would not be able to interact with ROP1 even when the mitochondria are recruited to the vacuole (Muta et al 1997 PMID: 9305894). Others have asserted that there is an additional cytosolic pool of C1QBP which can be recruited to the outer membrane of the mitochondria, thus allowing interaction with ROP1 immediately following rhoptry secretion into the cytosol or at the PVM (Xu et al 2009 PMID: 19164550). From these IFAs we are therefore unable to draw any firm conclusions.

We attempted proximity biotinylation to validate this potential interaction *in cellulo*, but C-terminal fusion of TurboID to ROP1 caused mislocalisation of the protein and prevented secretion of ROP1 to the parasitophorous vacuole. Based on the current evidence, we are unable to exclude that the interaction is an *in vitro* artefact as a result of cell lysis during the immunoprecipitation, and we clearly state this in the discussion. However, given this pulldown data is highly reproducible and technically sound, we believe it is important to include this result in the manuscript.

The authors state in their text that "Enhanced restriction of Δrop1 parasites is primarily mediated through increased vacuole destruction". Their data is more suggestive of growth restriction. The authors should provide more direct data for destruction of the vacuoles or change the wording to indicate it is due to growth restriction.

In the absence of specific results for vacuole destruction, which is technically challenging to determine quantitatively, we concluded by a process of elimination that restriction of ΔROP1 parasites at low MOI mostly likely occurs primarily through vacuole destruction, as we did not see strong evidence for vacuole size reduction and (now added in revision) do not see differences in parasite-induced host cell death. Reviewer #1 agrees in an earlier comment that vacuole destruction is the most likely mechanism. We note some subtle indications of strain- and host species-dependent differences, namely that RHΔROP1 parasites in THP-1 macrophages appear to have reduced vacuole size compared to RHΔUPRT (but not the complemented strain), but in all other cases the most likely mechanism

consistent with our data is destruction of vacuoles. We have revised our conclusions to reflect that this is an inference from the data rather than a definitive finding.

The authors should provide high resolution IFA images and decrease their size to an equivalent size of other sub-figures. Empty white space between sub-figures can be minimized. Font size of the figure labels/axis/titles should be matched and increased slightly.

Thank you for these suggestions. Each IFA image is only 2x2 cm so we feel that decreasing their size further would make them difficult to see.

Reviewer #2

Major Comments

The title indicates a positive role for ROP1 in subverting innate immune restriction, but the data indicate that a deficiency in ROP1 causes susceptibility to innate immune restriction. This might seem like a subtle discord, but in the absence of identifying the mechanism of ROP1 subversion of innate immune restriction it seems more appropriate to provide a title that better reflects the findings i.e., that ROP1 deficient parasites are more susceptible to innate immune restriction.

We recognise this point and have changed the title to reflect both this and new results added in revision: "*Toxoplasma gondii* virulence factor ROP1 reduces parasite susceptibility to murine and human innate immune restriction"

The reference strains and complement strains lack UPRT, but from the available information it appears the KO strains have UPRT. If this is the case, it is necessary to rule out that the presence of UPRT doesn't render parasites more susceptible to IFNg mediated killing by performing additional experiments comparing RHΔku80 with RHΔku80Δuprt and PruΔku80 with PruΔku80Δuprt.

This is correct, the reference and complemented strains lack UPRT while the ROP1 knockout strains have a functional UPRT. For the high-content imaging assay it was necessary to use a reference strain that expressed a fluorophore, as the knockout and complemented strains do, to allow for accurate segmentation of the parasites. We considered it preferable to insert the mCherry fluorophore at the well-established, non-essential UPRT locus rather than integrate it randomly. Complementation at the UPRT locus is widely used in the literature with no impact on virulence phenotypes: Shen et al 2014 (PMID: 24825012) Fig 4F, Wang et al 2020 (PMID: 33067458) Fig 5F, Wang et al 2020 (PMID: 31908049) Fig 5, Fox et al 2019 (PMID: 31266861) Fig 5, Olias et al 2016 (PMID: 27414498) Fig 6. Moreover, the

genome-wide CRISPR knockout screen of Wang et al 2020 (PMID: 33067458) has also demonstrated that knockout of UPRT does not affect growth of RH parasites in IFNγ-stimulated vs. naive BMDMs. UPRT has 100% amino acid identity between RH and PRU so no functional differences are expected.

Although it is understandable why the authors chose MEFs to test the role of C1QBP because MEFs were used for the Co-IP, the MEFs do not appear to be responding to IFNg for parasite growth restriction (-/+ IFNg % survival is at or above 100% for WT MEFs). As such, the authors are potentially blind to the role of C1QBP in the context of IFNg restriction. It would be ideal to repeat these experiments using BMDMs from WT and C1QBP KO mice to assess the potential contribution of C1QBP during IFNg restriction of T. gondii growth and survival.

We agree that it would be preferable to use ΔC1QBP BMDMs for these experiments. However, knockout of C1QBP is lethal in mice (Yagi et al 2012, PMID: 22904065), therefore it is not possible to obtain primary ΔC1QBP BMDMs. We also attempted siRNA-mediated knockdown of C1QBP in wild-type primary BMDMs using a pool of three different siRNAs, but were only able to achieve <10% knockdown of C1QBP. Given the immortalised MEFs we used here do not appear to be restricting parasite growth, we have decided to remove any conclusions relating to this experiment and include the data only in supplementary figures for the reference of other researchers working on C1QBP who may be using these cell lines (please also refer to our response to Reviewer #1 regarding this).

Minor Comments

Much of the data is analyzed with a paired two-sided t-test, but the authors used Bonferoni correction in some cases and Benjamini-Hochberg adjustment in other cases. It would be helpful to either consistently use the same correction or explain in a short section on stats in the methods the rationale for using different corrections.

We have changed the manuscript to consistently use Benjamini-Hochberg correction for all tests. These correction methods represent different approaches to the multiple-testing problem: Bonferroni correction controls the family-wise error rate, while the Benjamini-Hochberg procedure controls the false-discovery rate. We favour the FDR-based approach as it is less dependent on the somewhat-arbitrary decision as to what constitutes a “family” of tests - for example: should tests in the RH and PRU strains be in the same family; should tests for number of parasites and number of vacuoles/host cells be in the same family; should the tests in BMDMs vs. THP-1s should be the same family. At the alpha level of 0.05, one in twenty

significant results are false positives following Benjamini-Hochberg adjustment, as opposed to one in twenty of all tests carried out prior to adjustment. We find this appropriate for our study.

The IFA images in Figure 2B appear to show considerable redistribution of ROP1 from the rhoptries to other parts of the parasite upon short Trition-X100 treatment of formaldehyde fixed samples. Inclusion of a low concentration of glutaraldehyde might help preserve the normal distribution of ROP1. Alternatively, or additionally, permeabilization with saponin or digitonin could help visualize ROP1 associated with the PVM. Improving the imaging is not critical to support the conclusions of the study, but would nevertheless be an asset.

The ROP1 immunofluorescence staining within the parasites is likely from protein that is being trafficked through the ER or Golgi. This staining is more prominent with shorter Triton permeabilisation and in the complement lines. We have also observed PVM localisation of ROP1 using permeabilisation with 0.1% saponin for 15 min, but find that it is clearer and more consistent with the short Triton permeabilisation. We have added additional examples to the supplementary figure validating ROP1 knockout and complement.

Reviewer #3

Major Concern

The IFNg-dependent T. gondii control data as well as the Irgb6 recruitment data are extremely variable and preclude drawing any solid conclusions (Fig 2c-f, 3d, 4e-f, S3a-d, S8). The authors normalize the data by presenting the ratio of +IFNg/-IFNg in % of the measure they are analyzing. However, this does not "clean up" the data. The underlying cause for this problem are the extremely varied input, the time point analyzed and the nature of the microscopy experiment.

We have found that parasite IFNγ-restriction assays are highly variable between biological replicates by all methods that we have tried. We thank Reviewer #1 for sharing their similar experience, as we have heard the same from several colleagues. As it becomes more common to plot individual data points rather than summary statistics, this is increasingly apparent in the literature (e.g. Wang et al 2020 Fig 2, Rinkenberger et al 2021). While we could select the three “best” replicates to publish or instead present only summary statistics to obscure the variation present, we do not feel that this would be in the best interest of the field or of open science. We address Reviewer #3’s specific concerns below.

Presenting the data as a ratio/percentage of IFNγ-stimulated versus naive cells is not an attempt to "clean up" the data, it is standard practice in the field and reflects the hypothesis being tested. For example: Wang et al 2020 PMID: 33067458 (Saeij lab), Matta et al 2019 PMID: 31413201 (Sibley lab), Gay et al 2016 PMID: 27503074 (Hakimi lab), Bando et al 2018 PMID: 30283439 (Yamamoto lab), Fleckenstein et al 2012 PMID: 22802726 (Howard lab).

- Varied input: looking at the Supplementary tables and calculating the input MOI in the -IFNg samples for the BMDM, for example, they range from 0.1-6. Most of the MOIs are in the 0.1-1 range, but this is still a huge variation and not the MOI of 0.3 the authors are aiming to add. Input host cells within one experiment often differ 5 fold between the T. gondii strains analyzed. Consistency in input is not achieved.

The inputs of the restriction assays, as estimated by the parasite and host cell numbers in the unstimulated cells, are variable but not so extreme as Reviewer #3 says. Importantly, variability between strains within a biological replicate is far less than between biological replicates, and it is for this reason that we have analysed the results with paired tests. It is in fact a feature of microscopy-based assays that these sources of variability can be identified. We have attempted to be as open as possible with our data in reporting all of these measured parameters.

MOI is the most appropriate measure of inter- and intra-replicate variability as it accounts for both host cell and vacuole number. We have now added estimated MOI as a column in the supplementary data files (S2B and S3B: MOI = "Vacuole - Minus IFNG Median"/"Host Nuclei - Minus IFNG Median"). Out of 49 data points, there are only three instances in the BMDM restriction assays where MOI >1 (1.26, 1.06, 1.42) and none in THP-1s. Because the MOIs are low, the range of MOI within each replicate is small. Furthermore, for MOI ≤1, we expect there will be little qualitative effect on restriction as the host cells will be infected with either 1 or 0 vacuoles. In our data, we do not find any correlation between MOI and restriction for any individual strain or for all strains combined.

Regardless of the inter-replicate variability, the results for parasite restriction are statistically significant using an appropriate paired test. Our control parasite line PRUΔGRA12 behaves as expected (Fox et al 2019 PMID: 31266861) and differences between the RH and PRU strain in BMDMs dependent on the polymorphic effector ROP18 are apparent, although not tested here. While there is variation in the magnitude of restriction between replicates, there is a qualitative decrease in parasite survival in terms of total parasite number (Fig 2C and 2E) for ΔROP1

parasites compared to both ΔUPRT and the complemented lines in every replicate for both strains in BMDMs (7/7 replicates for each strain), and in all but one replicate in THP-1s (6/7 for RH, 7/7 for PRU). We consider that this restriction phenotype is highly robust.

- Time point analyzed: the authors chose to analyze 24h p.i. IFNg-stimulated MEFs, BMDMs and THP-1s. All will have undergone a significant amount of cell death at this time point. The authors even present the varying host cell numbers in Fig S3: the +IFNg cell numbers vs -IFNg cell numbers in BMDMs range from 35-70% for example in RH; THP-1 seem slightly better but still there is a range of 70-120%.

24 hpi or later is a standard timepoint at which to measure parasite restriction, as at earlier timepoints the dynamic range of the assays are reduced due to lower parasite numbers and therefore lower fluorescence, luminescence, or uracil uptake. For example, the following references all measure parasite restriction at at least 24 hpi: Wang et al 2020 Fig. 2: 24 hpi; Matta et al 2019 Fig. 3: 36-40 hpi; Gay et al 2016 Fig. 8: 36-48 hpi; Bando et al 2018: 24 hpi; Fleckenstein et al 2012 Fig. 4: 48 hpi.

The Howard lab has shown that host cell death is directly proportional to MOI in murine cells (Zhao et al 2009 PMID: 19197351, Lilue et al 2013 PMID: 24175088). At low MOIs of <1, as in our restriction assays, we would not expect enough parasite-induced cell death to explain the ~40% loss of BMDMs that we observe. We noted that some host cell loss was apparent even before infection as a result of interferon-reactive cell death, which was variable between batches of BMDMs derived from different donor mice. We observed less, and less variable, host cell death in THP-1 cell line-derived macrophages. Please refer to further comments on host cell death below.

- Nature of the experiment: Due to rampant host cell death at 24h p.i. analyzing the total parasite or vacuole number or even vacuole size is difficult in a microscopy experiment (dead cells will wash away even after fixation as they do not adhere anymore). Most laboratories (Howard, Yamamoto, Coers, Saeij, Steinfeldt etc), who study these mechanisms employ experimental systems that do not rely on washes/perturbations (plaque assays, plate reader T. gondii luciferase growth assays, uracil incorporation etc), or focus on the "number of parasites/vacuole" measure that are less dependent on host cell numbers (Saeij, Coers etc) or choose host cells that do not undergo IFNg-driven cell death (A549 cells, Sibley recent elife; only overexpression of the host factor induces cell death).

High-content imaging has previously been used to characterise IFNγ-dependent restriction of *T. gondii* (Gay et al 2016 PMID: 27503074 Fig 8D, Rinkenberger et al 2021 PMID: 34871166, Fisch et al 2021 PMID: 34931666) and for anti-parasitic drug screening (Touquet et al 2018 PMID: 30157171). Certainly different methods have different strengths and weaknesses: for example, fixed imaging-based methods required washing which will remove dead host cells from the analysis, whereas e.g. luciferase or uracil incorporation assays do not; however, with imaging we can measure multiple potential phenotypes simultaneously and identify potential confounding factors that would be unknown in other assays. Acknowledging that every assay has specific weaknesses, we verify the role of ROP1 by orthogonal methods: *in vivo* CRISPR screen for growth in the mouse peritoneum, *in vitro* growth in IFNγ-stimulated macrophages, and (now added in revision) *in vivo* virulence studies. The results of all of these assays concur.

If ROP1 alters the amount of host cell death induced in macrophages, host cell numbers in the +IFNg samples will vary in dependency of ROP1. Hence, in order to assess ROP1-dependent T. gondii restriction it is imperative to know if ROP1 alters host cell death. This can only be measured with cell death assays (total LDH or better a kinetic cell death assay) and not by looking at how many host cells remain after fixation in a microscopy experiment. To compare ROP1-driven parasite restriction, it is important to compare the same input MOI for each strain (-IFNg), determine a time point p.i. before host cell death has taken over and verify host cell numbers stay within a reasonable variation between strains. Input MOI consistency between T. gondii strains is typically measured by plaque viability assay.

We have now carried out kinetic propidium iodide uptake assays to determine formally whether ROP1 affects host cell death in BMDMs, which have been added to the revised manuscript. At an MOI of 0.3, as targeted in our restriction assays, there is very little parasite-induced cell death and no significant differences between parasite strains. This is in line with the results from the Howard lab mentioned above (Zhao et al 2009 PMID: 19197351, Lilue et al 2013 PMID: 24175088), and confirms that the majority of host cell loss observed in the high-content imaging assays at an average MOI of 0.3 is not parasite-induced.

We also carried out propidium iodide uptake assays at an MOI of 3, and found a slight but significant increase in host cell death for PRUΔROP1 in BMDMs of 10-15%. A small increase was also observed for RHΔROP1 compared to the complemented line, but was not significant compared to RHΔUPRT. This differential effect at low and high MOI is interesting, although we cannot rule out that a small phenotype is beyond the limit of detection at low MOI. We conclude that at low MOI host cell

death does not have a detectable role in the restriction of ΔROP1 parasites, leaving the most likely mechanism as disruption of vacuoles. At high MOI, vacuole breakage appears to result in host cell death as host cytosolic sensors recognise parasite-derived PAMPs: parasite DNA is sensed by AIM2 in activated THP-1 macrophages leading to apoptosis (Fisch et al 2019 PMID: 31268602), while in murine cells vacuole breakage by the IRGs leads to necrotic cell death (Zhao et al 2009 PMID: 19197351). Increased cell death at high MOI is therefore also consistent with increased disruption of vacuoles.

Other Major Comments

Fig 2C - Why are there datapoints with more than 100% T. gondii in +IFNg vs -IFNg samples? In these cases, no IFNg restriction was observed for the WT strain. This data is not reliable, as it has been demonstrated before that BMDMs control RH in an IFNg-dependent fashion.

This is one replicate out of seven. Although removing this replicate would not affect the statistical significance of the results in terms of total parasite number or number of vacuoles, we do not consider it appropriate to remove data because it does not match our expectations. Published data also show survival of RH parasites in BMDMs at or close to 100% in some replicates as a result of biological and technical variability (e.g. Wang et al 2020 PMID: 33067458, Fig. 2), which is to be expected in primary cells derived from different donor mice. The average survival we determine for RH across replicates in ~75%, which is in line with published data.

Fig 4C - The description of the IP in the legends and the materials is incomplete. In the materials it only states the loading of the IP fraction. Is the supernatant the post-IP fraction? 202200-HA is not described in the Figure legend. Why is the IP'ed version of C1QBP smaller than the supernatant version?

We will clarify this in the figure legend and methods. The supernatant is the post-IP fraction. It was necessary to load as much material as possible to detect C1QBP in the post-IP supernatant of the RHΔKU80 and PRUΔKU80-infected samples, but as the protein concentration in the supernatant is far higher than in the IP fraction this appears to have caused slight retardation of protein migration in these lanes. This is only apparent for C1QBP as it is the only protein detected in both supernatant and IP.

Fig 4E and F - There is almost no IFNg-dependent restriction in the WT MEFs for any of the parasite strains (4E). Some of the data even shows a dramatic increase of parasite load with IFNg versus without IFNg. Hence, no conclusion can be drawn about the function of C1QBP and

making a ratio of KO vs WT (Fig 4F) is not justified as there was no IFNg restriction to begin with in WT cells.

> We included these data as we found statistically significant differences between the C1QBP$^{flox/flox}$ and C1QBP$^{-/-}$ MEFs that were dependent on the presence of ROP1 in the parasites. However, after the concerns raised by all three reviewers we agree that it is better to not to draw conclusions from these assays given the lack of parasite restriction that would otherwise be expected in MEFs (e.g. Niedelman et al 2012 PMID: 22761577). We will include these data only in the supplementary figures as a reference for other researchers working on the role of C1QBP in innate immunity who may use these previously published cell lines.

Fig 3D - Something is wrong with the Irgb6 recruitment data. Many labs have shown that RH recruitment of Irgb6 is at most 10% due to the activity of ROP5 and ROP18. The authors get up to 40% recruitment for RH and again the data ranges from 5-42%, that's a huge variation!

> Recruitment of IRGB6 was determined by the ratio of the median fluorescence intensity in a 6 pixel radius around the parasites versus the median intensity in the rest of the infected cell cytoplasm. After carefully checking each step of this analysis pipeline, we found that the threshold for host cell cytoplasm segmentation based on CellMask staining was set too low and was including areas of empty space. This resulted in erroneously low median intensity in the host cell and so false-positive calling of IRGB6 recruitment to some vacuoles. We have corrected the cut-off threshold for host cell segmentation and reanalysed all the data with this corrected script. We now find average recruitment of ~5% for RHΔUPRT, ~15% for RHΔROP18, and ~60% for PRUΔUPRT (comparable to e.g. Fentress et al 2011, Fleckenstein et al 2012, Niedelman et al 2012, and Etheridge et al 2014), as well as less variability overall.

Fig S3A and C: The authors conclude that ROP1 does not affect vacuole size. I disagree. The data for THP-1 is significant. However, due to the noisy input with such varied MOI and varied host cell numbers, at the moment, no solid conclusion can be drawn.

> Although there is a significant difference between RHΔUPRT and RHΔROP1, this is not rescued by complementation so we are hesitant to claim this as a phenotype of ROP1. There are no significant differences in the PRU strain. We highlight this in the text of the results section, as it may be an important strain- or host species-dependent mechanism.

Fig S3B and D: The data clearly show huge variation in host cell numbers depending on IFNg and T. gondii infection. It is well-known that host cell death occurs in these experimental systems. In order to analyze whether ROP1 impacts host cell death a kinetic cell death assay is needed. Assessment of remaining host cell numbers in a 96 well plate microscopy experiment is not a quantitative assessment of host cell death, so the conclusion is not valid.

Please refer to comments on host cell death above.

Minor Comments

T. gondii "type" is always spelt with a lowercase "t" by convention.

We have corrected this.
