# Supplementary material for: Toxoplasma gondii virulence factor ROP1 reduces parasite susceptibility to murine and human innate immune restriction
Source: PLoS Pathog. 2022 Dec 7;18(12):e1011021. doi: 10.1371/journal.ppat.1011021 (PMC9762571; doi:10.1371/journal.ppat.1011021)
Supplement: Attachment — Submitted filename: Response to Reviewers 221117.pdf [file ppat.1011021.s019.pdf]

We thank all reviewers for their feedback and suggestions. Responses to individual points raised by the reviewers are detailed below.

Part I - Summary

Please use this section to discuss strengths/weaknesses of study, novelty/significance, general execution and scholarship.

Reviewer #1*: The authors have addressed concerns raised by the previous reviewers by addition of new data and revision of the manuscript. I have no further concerns and feel this will be an important contribution for the community.*

Reviewer #2*: In this manuscript, the authors use a CRISPR screen of secreted factors to identify those that are important for immune evasion in the host. In addition to a number of known factors and an array of intriguing candidates, they identify ROP1 as an important regulator of both the human and murine immune response. They then demonstrate that the protein associates with host C1QBP, providing a potential mechanism of action. The authors have responded well to the comments from the previous review - specific comments are below.*

Reviewer #3*: In the manuscript "Toxoplasma gondii virulence factor ROP1 reduces parasite susceptibility to murine and human innate immune restriction" by Butterworth et al. a CRISPR screen was performed to identify novel Toxoplasma genes that determine in vivo virulence. This is a repeat of the in vivo screen the Treeck lab performed in their 2019 Nature Communications paper. However, in this screen 75 genes that were not previously assessed by the Treeck lab (61 genes when comparing previous Treeck lab and Saeij lab in vivo screen data) were added.*

From this screen novel in vivo virulence candidates were identified that were not previously studied in vivo (but see Major Comment 1). Of these ROP1 was further studied and shown to determine parasite resistance to IFNy in both murine and human cells. ROP1 immunoprecipitation data indicate ROP1 can interact with C1QBP. Interestingly, there were significant differences in parasite growth between C1QBPflox/flox and C1QBP-/- MEFs which were dependent on the presence of ROP1 in the parasites. This indicates that the ROP1-C1QBP interaction is relevant for parasite growth. However, confusingly IFNy did not restrict Toxoplasma in the C1QBPflox/flox MEFs. In my opinion this is likely due to the fact that these MEFs were immortalized. We have seen that immortalization of HFFs and MEFs strongly affects the IFNy restriction phenotype.

Overall, this is an interesting manuscript that shows ROP1 is a novel Toxoplasma virulence factor that likely functions through its interaction with C1QBP. A more definitive role of for the ROP1-C1QBP interaction in IFNy-mediated parasite restriction will likely require knocking out C1QBP in non-immortalized cells.

Part II – Major Issues: Key Experiments Required for Acceptance

Please use this section to detail the key new experiments or modifications of existing experiments that should be absolutely required to validate study conclusions.

Generally, there should be no more than 3 such required experiments or major modifications for a "Major Revision" recommendation. If more than 3 experiments are necessary to validate the study conclusions, then you are encouraged to recommend "Reject".

***Reviewer #1**: (No Response)*

***Reviewer #2**: None, major issues previously raised were addressed in this submission*

***Reviewer #3**: No new experiments are required. However, I recommend they rewrite the result section of "in vivo virulence genes" and remake figure 1B (see Major Comment 1).*

Major Comments:

1) The main goal of this screen was to identify Toxoplasma genes that determine fitness in vivo but not in vitro. However, many of the genes that are claimed to be in vivo fitness genes already have large fitness effects in vitro based on data from genome-wide CRISPR screens performed by other labs (Lourido lab (Sidik et al 2016) and Saeij lab (Wang et al. 2020). Although the in vitro scores have a high Pearson correlation with previously published in vitro fitness scores (e.g., Pearson correlation with Sidik et al. is 0.54 indicating the reproducibility with previous CRISPR screens) the actual in vitro fitness scores are compressed and all scores are between -0.5 and 0.5. This indicates that the sgRNAs generated mutant parasites are not depleted in vitro after the 5 days of passaging. This is likely because of the smaller library size compared to the genome-wide libraries used in previous screens. However, this leads to the artificial identification of "in vivo" fitness genes that in fact already have a strong phenotype in vitro and are therefore not in vivo fitness genes but in vitro fitness genes.

A good example of this is GRA1, which is an essential gene (Rommereim et al. 2016). However, in this manuscript it is shown with an in vitro fitness of -0.2 while in Sidik et al. it was -5.3 and in Wang et al it was -6.6 (note that these are Log2FC scores and therefore the difference with -0.2 is extremely large!). Similarly, RASP2, GRA39, 205190, 301270, CLPTM1 and some others are genes already have significant in vitro fitness defects (see Sidik et al. and Wang et al.). When reanalyzing their data, now using the Sidik et al. passage 3 in vitro scores, and the Wang et al. passage 8 in vitro scores, it seems now only GRA2, GRA4, GRA25, GRA5, ROP1, GRA45, CDPK3, ROP18, and GRA12 are putative in vivo virulence genes (in vivo score <-1 in this screen and score >-1.5 in Sidik et al . passage 3 and Wang et al. passage 8). Of these only ROP1, GRA45 and CDPK3 were not previously included in the in vivo CRISPR screens of Young et al. and Sangare et al. CDPK3 (Wu et al 2022) and GRA45 (Wang et al. 2020) have already been studied in vivo. Therefore, ROP1 is the only novel identified gene from this screen. Overall, I suggest they remake Figure 1B using previously published in vitro fitness scores and rewrite the section discussing the in vivo CRISPR screen hits. Preferably they would use the Wang et al. in vitro passage 8 scores as the additional 5 days in vivo should be compared to an additional 5 days in vitro (which might correspond better to the Wang et al. in vitro passage 8).

We cannot fully explain the low dynamic range of the “in vitro” part of our screen, even compared to our prior screen (which had an *in vitro* range of -2 to +1), and subsequent screens we performed in our lab. Although these scores correlate well with prior datasets (added to Figure S1), as we are not able to filter out known essential genes with these scores it is a reasonable suggestion to further filter “hits” based on prior *in vitro*/HFF fitness phenotypes.

Some caution may be appropriate in extrapolating HFF fitness phenotypes from RH to PRU; furthermore, we note that the Wang et al HFF fitness scores skew highly negative, with a median L2FC of -2.78 compared to -0.66 in Sidik et al. In order to draw equivalent cutoffs across both datasets, we propose transforming the HFF fitness scores from Wang et al and Sidik et al to z-scores and filtering out any genes with a z-score of -1 in either dataset from the list of “hit” genes in with *in vivo* L2FCs ≤ -1 in our screen. This is equivalent to removing genes with HFF fitness scores more than one standard deviation below the mean in either dataset. This filtering effectively removes clearly essential genes such as GRA1, RASP2, and MyoF, while retaining e.g. TGME9_289150, for which we previously generated a knockout in Young et al 2019 that did not have a noticeable *in vitro* growth defect (though not formally quantified in that paper).

2) The authors state “Seven out of 150 unambiguous orthologues present in both screens had L2FCs of less than -1 in both screens, indicating that they are important for immune evasion of both the RH and PRU strains.” This is not entirely correct. ROP1 had a naïve BMDM fitness score of 1.13 and an IFNγ fitness score of

-0.17 in the Wang et al. screen. Although it is correct that the difference between naïve and IFNγ fitness <-1 because the IFNγ fitness score (which was IFNγ vs. plasmid input) is only -0.17, ROP1 was not identified as important for fitness in IFNγ-stimulated BMDM because the fitness score in IFNγ-stimulated BMDMs was >-1. Because of that it was not listed as a hit in the Wang et al. paper. Furthermore, the P-value was also not significant.

In this figure we used "Mean fitness (IFNγ vs. Naïve BMDM)" from Supplementary Data 1 of Wang et al in order to identify genes with an IFNγ-dependent phenotype independent of an overall *in vitro*/HFF growth fitness defect. We have revised the figure and results text to more clearly state that the fold change we use here is +/-IFNγ, and to caution that the result for ROP1, while interesting in light of the results of this paper, was not significant.

3) The claim that ROP1 prevents vacuole disruption is not substantiated by data. It is also possible it affects early parasite egress. In fact, the host cell death with high MOI seems to be consistent with early parasite egress.

Early egress is certainly a potential explanation for increased host cell death at high MOI. However, as with another alternative hypothesis that ROP1 directly inhibits host cell death in some manner, it is difficult to reconcile how a ~10% relative increase in cell death (e.g. for RHΔROP1 vs. RHΔUPRT) could account for a ~40% relative decrease in the number of vacuoles.

We note that in both THP-1s (Fisch et al 2019) and BMDMs (Zhao et al 2009), vacuole disruption is followed by host cell death. We therefore propose our favoured hypothesis that ΔROP1 vacuoles are disrupted at a higher rate, causing the reduced parasite/vacuole number we see by high content imaging, and that vacuole disruption leads to host cell death at some rate <100% (which may depend on MOI).

Our current data does not allow us to definitively distinguish between these hypotheses. We have revised the results and discussion sections to leave open these possibilities.

Part III – Minor Issues: Editorial and Data Presentation Modifications

Please use this section for editorial suggestions as well as relatively minor modifications of existing data that would enhance clarity.

***Reviewer #1**: (No Response)*

***Reviewer #2**: 1. Importantly, the authors show that PRUdeltaROP1 parasites are avirulent in vivo. However, the phenotype is not completely rescued by*

complementation (Fig 2C and in weight loss in 2D). In addition, complementation is not addressed at all in the text. While this is not problematic (many knockouts show incomplete recovery in Toxo), it should be discussed in the results. Is this due to the C-terminal HA tag or subtle levels of the protein in the complemented line?

Despite one PRUΔROP1::ROP1-HA-infected mouse surviving until the end of the experiment, there was no significant difference in survival for this strain compared to the wild-type PRUΔKU80. Although we have not currently included it in the manuscript, if we test for a difference in weight loss at day seven (the last day all mice were alive), there also is no difference between PRUΔKU80 and the complemented strain (although there are significant differences between PRUΔKU80 and ΔROP1 and between ΔROP1 and the complemented line). We therefore cannot strictly say that rescue is incomplete, as we cannot statistically detect a difference between the ΔKU80 and complemented strains (with the current five mice per group). Nevertheless we will add a sentence to highlight that one PRUΔROP1::ROP1-HA-infected mouse survived, potentially indicating incomplete rescue caused either by the HA tag, or slightly reduced parasite inoculum e.g. due to a clogged needle.

2. The link between ROP1 and C1QBP remains questionable and may be an artefact of these charged proteins interacting in lysates only – however the authors do clearly state this possibility and leave it as a candidate interactor which can be functionally resolved with additional studies.

We agree, and state this already in the discussion.

3. The authors make the statement "Since ROP1 from both strains can pull down C1QBP, we infer that this variant repeat region is likely not involved in the interaction". I don't think that the variable number of repeats means this region is any less likely to be involved in the interaction.

We have removed this sentence.

Reviewer #3: Minor comments:

1) They mention "We obtained such scores for 164 genes after filtering to remove genes with fewer than three well-represented sgRNAs (Supplementary Data 1)." It was unclear to me what exactly they mean by this. If a Toxoplasma gene is highly essential in vivo wouldn't all the sgRNAs targeting this gene disappear when the parasites are grown in vivo? Or do they mean with fewer than three well represented in the plasmid pool? Please specify.

We found in this screen that complete loss of sgRNAs from the population (zero or near-zero reads), is often associated with low representation in the starting plasmid library rather than the target genes being highly fitness-conferring. The starting

representation of sgRNAs in our plasmid library is not equal between guides (we think due to assembly from ssDNA secondary structure or dimer formation affecting cloning efficiency), and so sgRNAs which are initially poorly represented can be easily "lost" from the parasite population independently of a true fitness defect. Including these in the analysis increased the variability between sgRNAs targeting the same gene, and in some cases resulted in spuriously high L2FCs that did not correlate with published datasets.

We first tried adding pseudo-counts to the data, but this still resulted in inflated fitness phenotypes. We therefore filtered the sgRNAs to only include for analysis those for which are detected with at least 50 raw reads in every sample throughout the screen. While this strategy may bias our results to less-strong fitness scores, we prefer this to potentially allowing a lot of false positive hits. We still retain genes with large L2FCs (e.g. GRA12, ROP18, RASP1) and can be more confident that these L2FCs are real and not the result of a coverage bottleneck. We also note that, if we skip this filtering step, the median *in vitro* L2FC for the essential control gene GRA1 is still only -0.36, thus this filtering strategy does not explain the overall low *in vitro* fold changes we find in this screen.

> *2) I'm not sure if I am misinterpreting the mass-spec suppl data but only 5, 5, and 1 ROP1 peptides were identified in the 3 IP experiments. It seems not a lot of ROP1 was immunoprecipitated and it is a bit strange that more C1QBP is immunoprecipitated than the actual ROP1.*

It is correct that relatively few unique peptides from ROP1 are detected, although these are specifically detected in the ROP1-HA samples and the difference in iBAQ (a measure of total protein abundance) is large compared to the KU80 samples. The low number of peptides is broadly in line with other proteomic datasets (available on ToxoDB), which is likely because tryptic digestion of ROP1 produces mainly peptides that are very long or very short and not well suited to MS/MS identification.

Crystal structures of C1QBP indicate that it is a trimeric protein. We hypothesise that a single ROP1 molecule interacts with a C1QBP trimer, giving a 1:3 molar interaction and potentially accounting for higher enrichment of C1QBP compared to the bait ROP1.

> *3) The authors seem to focus on the ROP1 PVM localization. However, I suppose ROP1 is also localized in cytoplasm when rhoptry bulb contents are secreted into the host cytoplasm. However, this might be difficult to visualize.*

We have not seen ROP1 or ROP1-HA staining in the cytoplasm, but it may indeed be that the signal is too diffuse to detect at later timepoints. We have added a sentence to the discussion stating that ROP1 is also detected in evacuoles in the host cell cytoplasm immediately after invasion (Håkansson et al 2001).
